# The Genome-Wide Identification and Expression Analysis of the AMT Gene Family in Foxtail Millet

**DOI:** 10.3390/biology15090710

**Published:** 2026-04-30

**Authors:** Mingge Han, Junliang Yan, Kang Zhao, Yang Zhang, Xiaojing Li, Mengyu Xue, Juwu Gong, Yajie Ma

**Affiliations:** 1 Anyang Academy of Agricultural Sciences, Anyang 455000, China; 2 Cotton Research Institute, Chinese Academy of Agricultural Sciences, Anyang 455000, China

**Keywords:** bioinformatics, gene expression, NaCl stress, *Setaria italica*, SiAMT

## Abstract

Ammonium transporters (AMTs) represent a class of proteins within the ammonium transporter domain, which play an important role in mediating the transmembrane transport of NH_4_^+^ in plants. However, research on AMT genes in foxtail millet remains limited. In this study, members of the AMT gene family in foxtail millet were identified at the whole genome level through bioinformatic analysis. The gene structure, evolutionary relationships, chromosomal localization, interspecies collinearity, *cis*-acting elements, and expression patterns of SiAMT members were systematically analyzed. The results revealed that there were nine SiAMT family members in foxtail millet, with molecular weights ranging from 49.5 to 53.8 kDa. Phylogenetic analysis classified them into three groups, which were unevenly distributed across chromosomes. The analysis of promoter *cis*-acting elements identified multiple regulatory elements, including light-, anaerobic-, and hormone-responsive elements. Collinearity analysis showed that the divergence time of AMT family members in foxtail millet and rice was more recent compared to *Arabidopsis thaliana*. The expression levels of SiAMT members varied across different tissues of foxtail millet, with most SiAMT family members showing high expression in roots, while *SiAMT7* was significantly expressed in leaves. qRT-PCR analysis showed that *SiAMT1* was significantly down-regulated in roots, stems, and leaves under salt stress. This study provides a theoretical foundation for further investigation into the functions of the AMT gene family.

## Simple Summary

This study aims to identify and analyze the sequence characteristics of AMT gene members in foxtail millet using the foxtail millet genome database and bioinformatic methods and to probe the potential biological functions of the AMT genes in foxtail millet. A total of nine AMT family members were identified. In addition, their gene structure was relatively conserved. The promoter elements included multiple light response, anaerobic response, hormone response and other functional elements. Most SiAMT family members were highly expressed in roots, while *SiAMT7* was significantly expressed in leaves. This study provides a theoretical foundation for molecular breeding by further exploring AMT genes.

## 1. Introduction

Nitrogen is a crucial nutrient for plant growth and development, playing a decisive role in determining crop yields [1]. However, due to its low availability in soil [2], over 110 million tons of nitrogen fertilizer is required annually. Excessive nitrogen application wastes energy, increases agricultural costs, and causes environmental pollution [3,4,5]. NH_4_^+^ and NO_3_^−^ are the two primary forms of nitrogen absorbed by plants from soil [6]. However, NH_4_^+^ is the preferred nitrogen source for root absorption, particularly in nitrogen-deficient plants [7,8], because it has lower absorption and assimilation energy requirements compared to NO_3_^−^.

The transmembrane transport of NH_4_^+^ by plant roots is primarily mediated by ammonium transporters (AMTs) located on the plasma membrane [9,10]. Kinetic experiments have revealed two classes of ammonium transporters: high-affinity (HATSs) and low-affinity (LATSs). These two types work together to dynamically regulate ammonium uptake throughout plant growth and development [11,12,13]. When ammonia concentrations are sub-millimolar, plants primarily utilize high-affinity AMTs for ammonium absorption, while low-affinity AMTs are activated at millimolar concentrations [14,15]. However, the concentration of ammonium ions in the soil is usually less than 1 mM [16]. Accordingly, the high-affinity ammonium transport system constitutes the principal mechanism for ammonium nitrogen acquisition by plants.

*Arabidopsis thaliana* is the species from which the first plant ammonium transporter (AMT) was reported. Notably, the mRNA expression of *AtAMT1;1* correlates strongly with nitrogen status changes in plants [17]. Approximately 30–35% of ammonium uptake in nitrogen-deficient roots is attributed to *AtAMT1;1* and *AtAMT1;3* [18], while *AtAMT1;2* contributes only 18–26% [19]. In rice, the knockout of *OsAMT1;1*, *OsAMT1;2*, and *OsAMT1;3* reduced ammonium uptake by 95%, demonstrating the distinct regulatory mechanisms of these *AMT1* members. The AMT gene family is classified into subfamilies based on amino acid sequences, particularly the *AMT1* and *AMT2* subfamilies [10,20,21]. In sugarcane, *ScAMT2;1* enhances ammonium transport in rhizomes under high-ammonium conditions, while *ScAMT3;3*, a low-affinity transporter expressed in young and mature leaves, plays a key role in ammonium transport [22]. The high expression of *CsAMT2.2* and *CsAMT2.3* in roots indicates their crucial role in regulating ammonium uptake [23].

Aside from their function in nitrogen transport and uptake, some ammonium transporter (AMT) genes may also regulate the circadian clock and be involved in other biological processes. *AtAMT1.3* showed peak ammonium uptake at dusk, which then gradually decreased as light intensity dropped [8]. *LeAMT1.2* and *LeAMT1.3* in tomatoes also show rhythmic regulatory characteristics [24]. Twelve members of the AMT gene family were identified in the sweet potato genome, with *IbAMT1.3* and *IbAMT1.5* playing key roles in nitrogen utilization and storage root development [25]. *AMT1.1*, *AMT1.3*, and *AMT2.3* participate in the plant response to pathogens in both rice and wheat [26,27]. *VcAMT14* plays a key role in mycorrhiza-mediated nitrogen uptake [28]. *LjAMT2.1* and *LjAMT2.2* from *Lotus japonicus*, as well as *MtAMT2.3* from *Medicago truncatula*, may play a role in transport from the symbiotic host plant to nitrogen-fixing rhizobia and arbuscular mycorrhizal (AM) fungi [29,30,31]. *GmAMT2.1/2.2* alleviates cadmium stress by affecting the nitrogen nutrition pattern of soybeans [32]. Thus, AMTs likely play a crucial role in enabling plants to obtain adequate ammonium for growth, development, and adaptation to the environment.

Studies have found that when ammonium nitrogen is used as the nitrogen source, *Arabidopsis* seedlings exhibit lower sensitivity to salt stress, and this response is mediated by ammonium transporters (AMTs). Salt-stressed plants of the SOS2 mutant show lower ammonium uptake than wild-type plants. Conversely, the salt-induced activation of SOS2 kinase is enhanced by AMT-mediated ammonium uptake. SOS2 fine-tunes and maintains plant ammonium uptake by activating *AMT1;1*, thereby optimizing the plant’s response mechanism to salt stress [33]. Soil salinization poses a serious threat to agricultural production and plant development. Under elevated salt conditions, nitrification is suppressed, making ammonium (NH_4_^+^) the dominant inorganic nitrogen source for plants. Because AMTs mediate ammonium transport in plants, investigating whether AMTs are induced by salt stress is an important issue.

Foxtail millet (*Setaria italica* L.), as a new type of C4 model crop in the grass family, is also one of the main coarse grain crops in China, with outstanding stress resistance and high nitrogen utilization efficiency [34,35,36]. The publication of the high-quality genome of millet has provided an important genomic reference for millet gene research [37]. With the gradual completion of genome sequencing, millet has gradually become a model crop for functional genomic research [38,39]. Currently, six, sixteen, six, twenty, eight, and sixteen AMT genes have been identified in *Arabidopsis* [18], soybean [40], sugarcane [41], rapeseed [42], pepper [43], and poplar [44] crops, respectively. However, no studies on the AMT gene family at the genomic level have been conducted for millet.

To achieve this goal, the AMT gene family in the foxtail millet genome was identified and investigated using public data and bioinformatic approaches, covering phylogenetic relationships, gene and protein structure, *cis*-acting elements, and tissue-specific expression patterns. Given the strong stress tolerance of foxtail millet, this study also examined the expression patterns of AMT gene family members under salt stress. In this study, we aimed to provide a reference for a further functional analysis of the AMT gene family in foxtail millet and to establish a theoretical foundation for marker-assisted breeding.

## 2. Materials and Methods

### 2.1. Plant Material and Treatment

For this study, the three-leaf-stage seedlings of the foxtail millet variety Yugu 18 were used to analyze the expression of AMT gene family members under salt stress at the seedling stage. First, foxtail millet seeds were surface-sterilized and then sown in seedling pots containing nutrient soil. The seedlings were grown in a growth chamber with a photoperiod of 28 °C for 16 h of light and 25 °C for 8 h of darkness, with a humidity of 65%, until the three-leaf stage. For salt stress treatment, the three-leaf-stage seedlings were immersed in 200 mL of 200 mM NaCl solution. Seedlings treated with ddH_2_O served as the control. The treatment lasted for 9 h, after which samples were collected. Each treatment was repeated three times. All samples were frozen in liquid nitrogen and stored at −80 °C.

### 2.2. Species Database and Software Sources

The genomic data of *Arabidopsis thaliana*, grape, rice, and maize were obtained from the Plant Genome Database (https://phytozome-next.jgi.doe.gov/blast-search, accessed on 27 April 2026). The local BLAST + 2.17.0 alignment tool was downloaded from the National Center for Biotechnology Information (NCBI) website (https://www.ncbi.nlm.nih.gov/, accessed on 27 April 2026), and Hmmer domain retrieval software was downloaded from the website http://hmmer.org/download.html, accessed on 27 April 2026.

### 2.3. Identification and Analysis of AMT Gene Family and Its Physicochemical Properties

The protein sequences of six AMT family members in *Arabidopsis thaliana* were downloaded from the *Arabidopsis* website (https://www.arabidopsis.org/, accessed on 27 April 2026), and the HMM model of AMTs (PF00909) was downloaded from the Pfam online database. Members of the foxtail millet AMT gene family were identified using local BLAST+2.17.0 and HMMER-3.3.2 software. The results from both were combined, and the overlapping sequences were used for further analysis. These protein sequences were subsequently validated via the NCBI-CDD website. After removing incomplete genes, a total of nine SiAMT gene family members were identified in foxtail millet, named *SiAMT1* to *SiAMT9*, based on their chromosomal locations. Using the same identification method, we identified AMT gene family members in rice, *Setaria viridis*, maize, and other species, naming them according to their chromosomal positions. The physicochemical properties and subcellular localization of the SiAMT gene family members in foxtail millet were analyzed using the ExPASy (https://web.expasy.org/protparam/, accessed on 27 April 2026) and WoLF PSORT (https://wolfpsort.hgc.jp/, accessed on 27 April 2026) online tools.

### 2.4. Phylogenetic Analysis of AMT Gene Family

A total of 69 AMT genes were identified from foxtail millet, maize, rice, *Setaria viridis*, grape, cocoa, and *Arabidopsis.* Protein sequence alignment was carried out using the MUSCLE tool in MEGA X. Then, based on the Poisson substitution model and default parameters in MEGA X, a neighbor-joining (NJ) phylogenetic tree was constructed for these species, with a bootstrap of 1000 replicates; all other parameters retained their default settings.

### 2.5. Chromosomal Distribution and Collinearity Display

All SiAMT genes and SrAMT genes were mapped to S. *italica* and S. *viridis* chromosomes separately based on the physical location information. Visualization analysis was conducted using TBtools v1.0987663.

### 2.6. Conservation Protein Motif and Gene Structure Analysis of SiAMT Gene Family Members

In this study, the conserved motifs of AMT family members in millet were analyzed using the Multiple Em for Motif Elicitation (MEME) Suite 5.5.8 website (https://meme-suite.org/meme/tools/meme, accessed on 27 April 2026). The upper limit for motif detection was set to 8, while all other parameters were retained as defaults. The conserved motifs, evolutionary relationship, and gene structure were visualized using TBtools software, v1.0987663.

### 2.7. Promoter Cis-Acting Elements and Expression Patterns of SiAMT Gene Family Members

We used the TBtools tool to extract the 2000 bp DNA sequence upstream of the AMT gene from the foxtail millet genomic data as the promoter of the AMT gene for analysis. The *cis*-acting elements in the AMT gene promoter were predicted using the PlantCARE online website (http://bioinformatics.psb.ugent.be/webtools/plantcare/html/, accessed on 27 April 2026). The expression levels of AMT gene family members in various tissues were obtained from the Setaria-Db online database, and the acquired expression data were visualized using TBtools.

### 2.8. Total RNA Extraction, cDNA Synthesis, and qRT-PCR Analysis

We extracted total RNA from foxtail millet by employing the Plant Total RNA Kit (provided by Aidlab, Beijing, China) in accordance with the manufacturer’s specifications. The RNA integrity was evaluated -on a 1% agarose gel, followed by first strand cDNA synthesis using the EasyScript One-Step gDNA Removal and cDNA Synthesis SuperMix kit (TransGen Biotech, Beijing, China). The relative transcript levels of the SiAMT gene under salt stress was determined via qRT-PCR. The primer sequences were designed using an online resource (https://www.genscript.com/tools/real-time-pcr-taqman-primer-design-tool, accessed on 27 April 2026), and the relative expression was calculated using the 2^−∆∆ct^ method. The actin gene (Genebank number: AF288226) of foxtail millet was used as an internal control. qRT-PCR was performed using the Applied Biosystems@7500 Fast system and TransStart Top Green qPCR SuperMix (provided by TransGen Biotech, Beijing, China). For each sample, three replicates were performed, and the relative expression levels were quantified. All the primer sequences used in this study are listed in Supplementary Table S1.

### 2.9. Statistical Analysis

Data analysis was conducted using IBM SPSS Statistics V21.0, and histograms were drawn using GraphPad Prism 8.0. Student’s *t*-test was applied for comparisons, with asterisks marking significant differences versus controls (* *p* < 0.05, ** *p* < 0.01).

## 3. Results

### 3.1. Identification and Physicochemical Property Analysis of AMT Gene Family in Foxtail Millet

Based on the analyses using the local BLAST, HMM, and public databases, a total of nine AMT genes were identified, sequentially named *SiAMT1 to SiAMT9* according to their chromosomal distribution. Their physicochemical properties, including the coding sequence (CDS) length, protein molecular weight, pI, and subcellular localization, were determined. Amino acid sequence analysis revealed that the SiAMT family genes encode 465–504 amino acids, with SiAMT5 having the longest protein sequence (504 aa) and SiAMT9 the shortest (465 aa). Their molecular weights ranged from 49.47 (SiAMT9) kDa to 53.82 (SiAMT5) kDa, while the isoelectric points varied from 6.24 (SiAMT1) to 8.82 (SiAMT4). The average hydrophilicity index of the amino acid sequences was 0.420 (SiAMT6) to 0.592 (SiAMT9); all positive values indicated that these proteins were hydrophobic, though with varying degrees of hydrophobicity. WoLF PSORT analysis indicated that the SiAMT proteins were predominantly associated with the plasma membrane. In addition, the AMT protein was also predicted to be located on the vacuole and endoplasmic reticulum (Table 1).

### 3.2. Chromosomal Localization of AMT Family Members in Foxtail Millet

**Figure 1:**
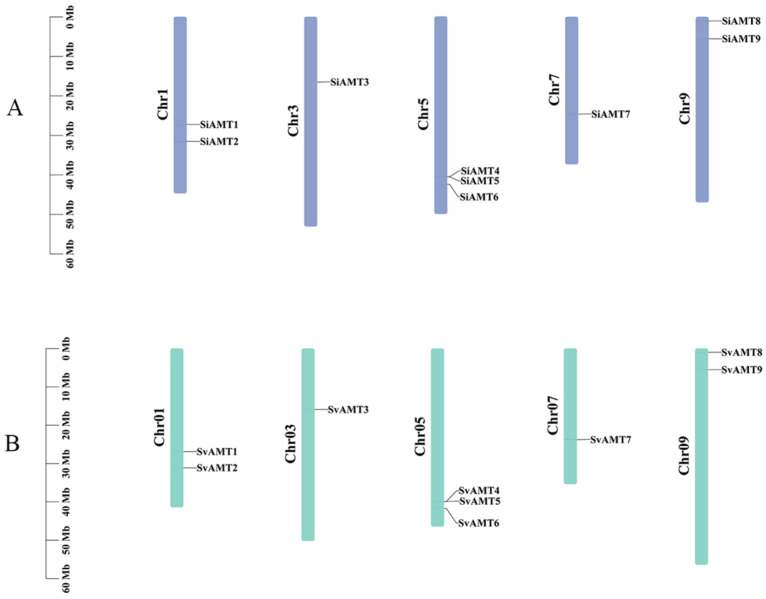
Chromosome distribution of the AMT gene family in foxtail millet. Note: (**A**) represents *Setaria italica*, and (**B**) represents *Setaria viridis*.

To gain a more intuitive understanding of the gene distribution on chromosomes, a chromosomal physical map of the AMT gene family members in *S*. *italica* millet and *S*. *viridis* was constructed based on the physical locations of each AMT member on the chromosomes. Figure 1 shows that these AMT genes were unevenly distributed across the chromosomes, primarily distributed on chromosomes 1, 3, 5, 7, and 9. *SiAMT5* and *SiAMT4* were tandem repeat genes. Different genes located on different chromosomes may be related to their functions. In *Setaria viridis*, the distribution of AMT genes on the chromosomes was the same as in foxtail millet.

### 3.3. Analysis of Gene Structure and Conserved Domain of AMT Family Members in Millet

**Figure 2:**
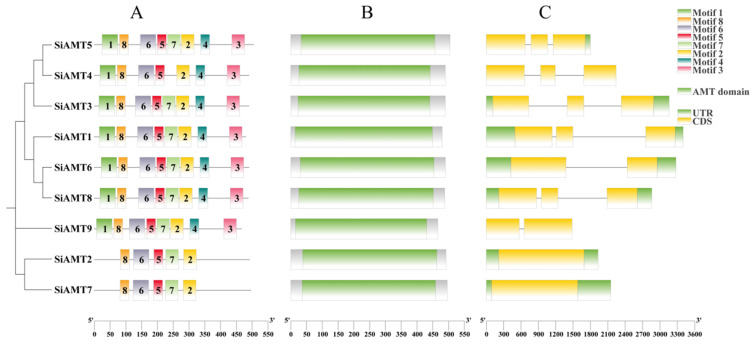
Analysis of conserved motif, domains, and exon-intron structures of AMT gene family members in foxtail millet. Note: (**A**) represents conserved motifs, (**B**) represents domains, and (**C**) represents exon-intron structure.

The gene’s function is closely related to its structure and conserved domains. We analyzed the evolutionary relationships, domains, and intron-exon structures of the AMT family genes (Figure 2). All members of the AMT family contained the AMT domain. Phylogenetic tree topology served as the basis for classifying the AMTs, and the resulting exon-intron distribution patterns were correlated with physiological functions. AMTs belonging to the same subfamily shared similar exon and intron structures, but the distribution of the gene structural motifs varied among subfamilies, suggesting that these genes may possess different functions. All AMTs contained five conserved domains, motif 8, motif 6, motif 5, motif 7, and motif 2, indicating that these were common structural elements in the AMT gene family.

### 3.4. Systematic Evolutionary Analysis of AMT Gene Family Members in Millet

**Figure 3:**
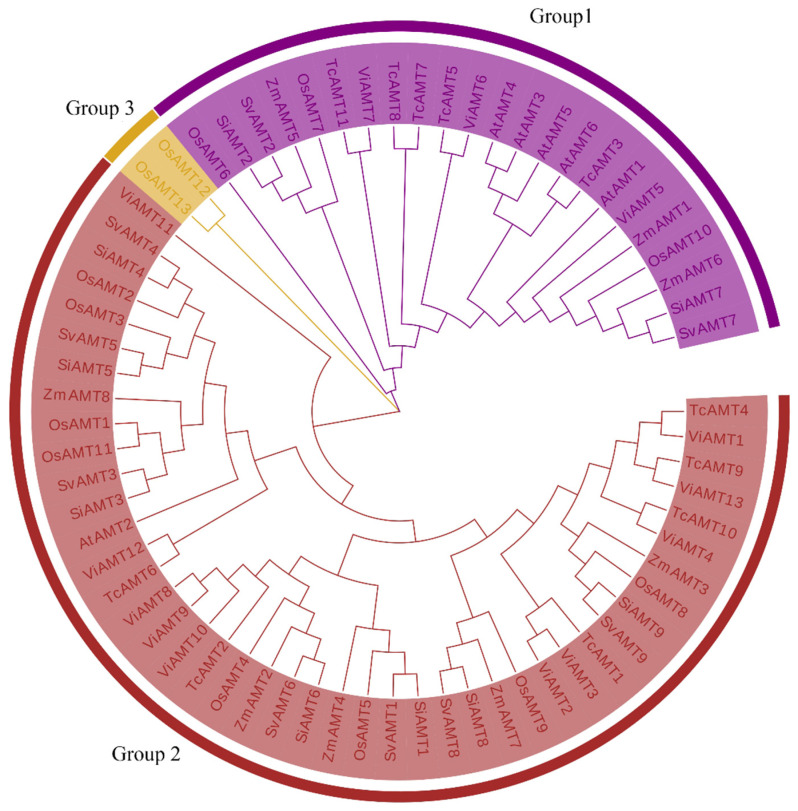
Evolutionary analysis of AMT gene family members in foxtail millet and other crops.

*Arabidopsis thaliana* contains six AMT gene family members. Based on their protein sequences and domains, these members were further identified in species including foxtail millet, rice, maize, cocoa, and grape. Specifically, 13, 9, 9, 11, 13, and 8 AMT family members were identified in rice, foxtail millet, *Setaria viridis*, cocoa, grape, and maize, respectively. To further elucidate the evolutionary relationships of SiAMTs, an evolutionary tree was constructed for the AMT gene family members from millet, foxtail millet, cocoa, rice, maize, grape, and *Arabidopsis* (Figure 3). Systematic evolutionary analysis revealed that the AMT family members are divided into three clusters (Group 1, Group 2, Group 3), with Group 1 containing 44 family members, Group 2 comprising two gene families, and Group 3 including 23 family members. Notably, millet contained the most AMT family members in Group 1. The branching of the evolutionary tree showed that foxtail millet was the most closely related to green foxtail, with the AMT gene branch of foxtail millet being immediately adjacent to that of green foxtail.

### 3.5. Gene Replication and Collinearity Analysis

**Figure 4:**
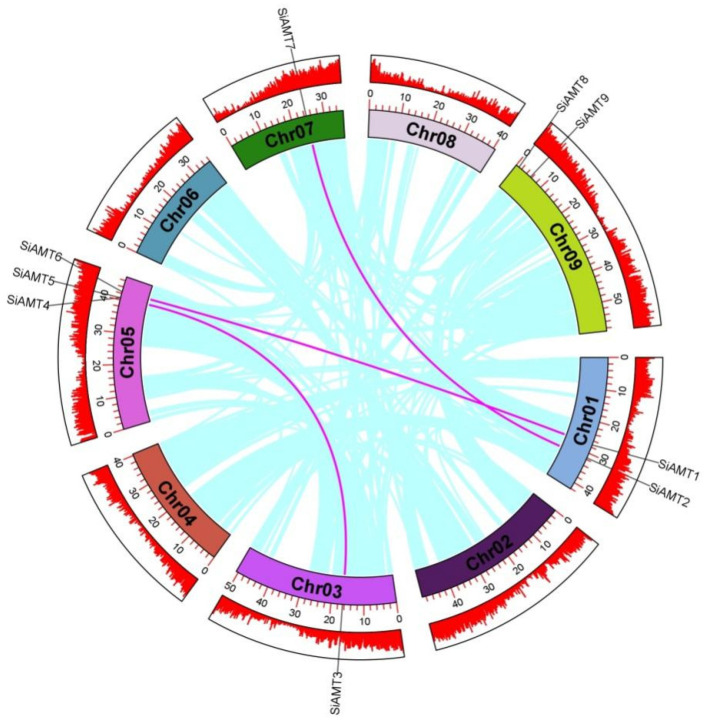
Synthetic connections of duplicated AMT genes across *Setaria italica.* The purple lines represent the gene pair.

Gene duplication events are recognized as crucial in the expansion of gene families. These events include whole genome duplication (WGD), segmental duplication, and tandem duplication. Large-scale WGD, small-scale tandem duplication, and segmental duplication can be identified through collinearity analysis, serving as key indicators for species evolution. To investigate the amplification mechanisms of the millet AMT gene family, a collinearity map of the SiAMT gene family was constructed. As illustrated in Figure 4, we used identical colors to connect lines belonging to the same gene. The analysis revealed that six members formed three pairs of segmentally duplicated genes (*SiAMT1* and *SiAMT6*, *SiAMT2* and *SiAMT7*, and *SiAMT3* and *SiAMT4*), accounting for 67% of the gene family members. Each pair belonged to the same subcategory, suggesting that these chromosomal segments may have undergone duplication events during evolution, with some remaining undifferentiated and potentially exhibiting functional redundancy.

**Figure 5:**
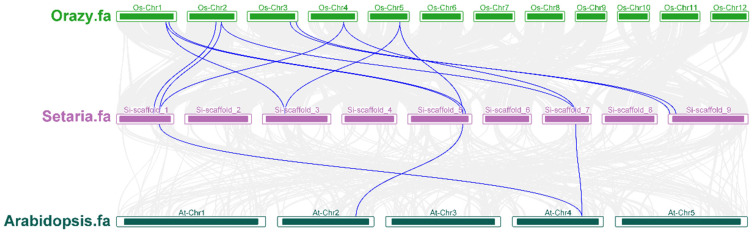
Collinearity analysis of AMTs in foxtail millet, rice, and *Arabidopsis*. The blue lines represent the gene pair.

Synteny analysis was conducted to investigate the evolutionary history of the SiAMT gene family. This analysis compared foxtail millet with *Arabidopsis*, a model dicotyledon, and rice, a model monocotyledon. The results revealed that foxtail millet shares only three AMT orthologous genes with the *Arabidopsis* genome, while it shares twelve with the rice genome (Figure 5). This indicates that the AMT gene family in foxtail millet diverged from rice more recently in evolutionary terms, whereas its divergence from *Arabidopsis* occurred relatively earlier.

### 3.6. Prediction of Cis-Acting Elements of AMT Promoters in Foxtail Millet

**Figure 6:**
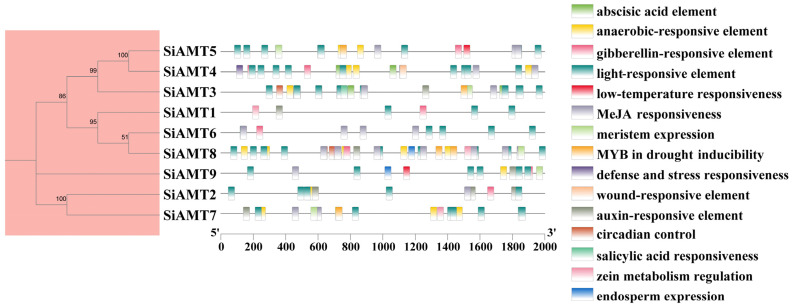
Investigation of *cis*-regulatory sequences in promoter region of AMT gene family.

To elucidate the potential transcriptional regulatory mechanisms of the SiAMT genes in foxtail millet, the *cis*-regulatory elements within the promoter sequences were predicted and analyzed. The results showed that the members of the foxtail millet AMT gene family contained 15 other types of functional elements, including light-responsive elements, plant hormone response elements, stress response elements, and growth/development-related response elements (Figure 6). Among the plant hormone response elements, auxin-responsive elements, abscisic acid-responsive elements, gibberellin-responsive elements, and jasmonic acid-responsive elements were identified.

### 3.7. Expression Pattern of AMT Genes in Different Tissues

**Figure 7:**
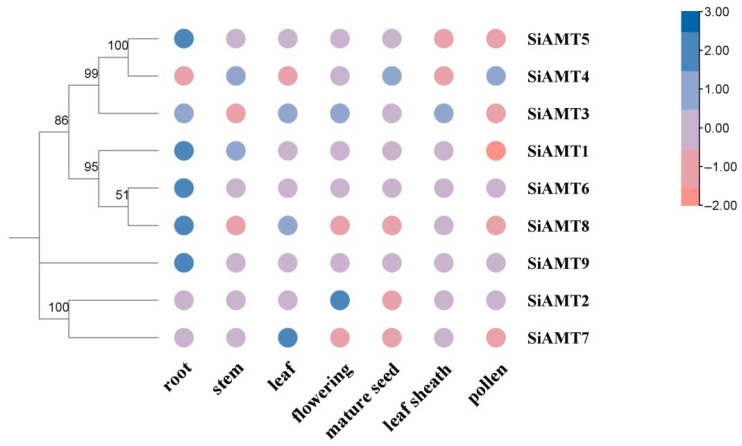
Heatmap analysis across different tissues of *Setaria italica*.

Using the foxtail millet online database (Setaria-DB database), transcriptome data for various developmental stages and tissues of Yugu 1 were downloaded to analyze their expression patterns. The results showed distinct expression profiles across different tissues. Specifically, *SiAMT5*, *SiAMT1*, *SiAMT6*, *SiAMT8*, and *SiAMT9* were significantly expressed in roots; *SiAMT7* was expressed in leaves; and *SiAMT2* was expressed in flowering tissues, while other genes exhibited no significant expression differences in other tissues (Figure 7). This indicates that members of the SiAMT gene family exhibit different expression patterns across various tissues of foxtail millet.

### 3.8. Expression Analysis of AMT Genes in Foxtail Millet Under Salt Stress

**Figure 8:**
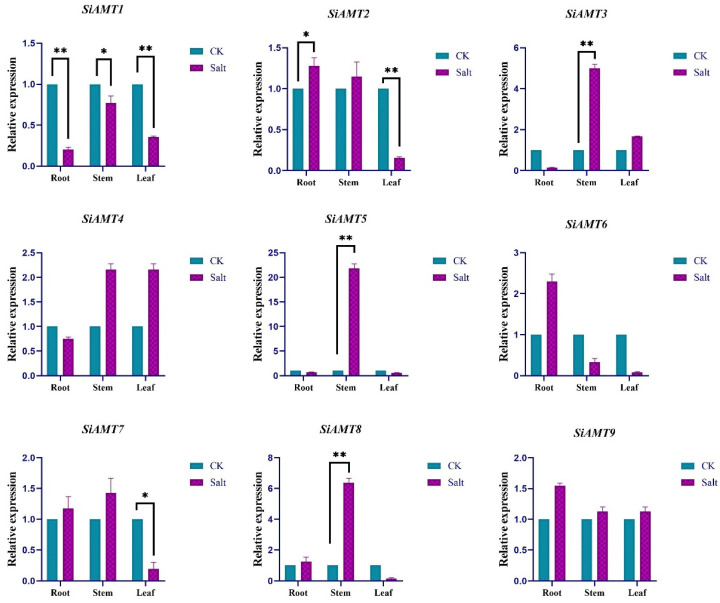
Relative expression patterns of SiAMT genes under salt stress. * *p* < 0.05, ** *p* < 0.01.

To validate the expression of SiAMT genes in response to salt stress, the expression profiles of individual family members were analyzed via qRT-PCR. The data revealed that *SiAMT1* and *SiAMT2* were significantly expressed in roots; *SiAMT1*, *SiAMT3*, *SiAMT5,* and *SiAMT8* were clearly expressed in stems; and *SiAMT1*, *SiAMT2,* and *SiAMT7* were markedly expressed in leaves. Among them, the *SiAMT1* gene was noticeably down-regulated in roots, stems and leaves under salt stress (Figure 8).

## 4. Discussion

Ammonium transporters (AMTs) serve a critical function in the absorption and transport of nitrogen. Extensive studies have identified and characterized the AMT gene family across various plant species, such as rice, wheat, and poplar [14,45,46,47,48]. However, the functional evolution mechanism of the SiAMT gene family members in foxtail millet and their expression under salt stress remain unclear. Based on the latest reference genome of foxtail millet, this study used bioinformatic methods to conduct the genome-wide identification and characteristic analysis of AMT genes. There were nine SiAMT genes in foxtail millet, the same number as in *Setaria viridis*. Reports state that foxtail millet originated from *Setaria viridis*, and as seen from their chromosomal distribution, these genes remained highly conserved throughout evolution. Moreover, from the branches of the phylogenetic tree, foxtail millet had a closer genetic relationship with *Setaria viridis*, which further confirmed that green foxtail (*Setaria viridis*) is the ancestor of foxtail millet (*Setaria italica*) [49]. All members of the AMT family contained the ammonium transporter (PF00909) domain; hence, these members may be related to ammonium transport.

Unlike the model plants rice and *Arabidopsis thaliana*, which have 6 to 13 AMT genes respectively, foxtail millet has an intermediate number, with nine members in its AMT gene family. In the phylogenetic tree, branch topology reflects both evolutionary relationships and genetic divergence among plant species. The AMT gene branch of foxtail millet is immediately adjacent to those of green foxtail, followed sequentially by maize and rice. In contrast, the branches of *Arabidopsis*, cacao, and other plants are positioned relatively farther away. The evolutionary analysis of SiAMTs with SvAMTs, AtAMTs, OsAMTs, and ZmAMTs shows that the results can be divided into three groups, which is consistent with the grouping of *Setaria viridis* and inconsistent with the grouping results of *Arabidopsis thaliana* and rice in the evolutionary relationships of other crops. This indicates that there may be significant evolutionary differences in AMTs among different crops, possibly due to different sequence duplications or deletions during the evolutionary process. The predicted subcellular localizations of these proteins include the plasma membrane, vacuole, and endoplasmic reticulum, indicating that these SiAMTs may function in directing ammonium ions to distinct subcellular regions.

Differences in gene structure can reflect the evolutionary relationship among gene families. In addition to variations in gene number and protein features, the SiAMT genes are also highly variable in gene structure. First, both *SiAMT4* and *SiAMT9* both lack UTRs, which may result from genetic variation or mutation leading to UTR deletion. Second, both the *SiAMT2* and *SiAMT7* genes lack introns. The intron number generally increases with the complexity of eukaryotic genomes and organisms [50,51]. Third, *SiAMT1*, *SiAMT3*, *SiAMT5*, *SiAMT6*, and *SiAMT8* all possess introns, CDSs, and UTRs. This is different from the result for GmAMT1 in soybean, which contains no introns [40]. Nevertheless, the lengths of these introns and UTRs differ among these SiAMT genes. In sum, 77.8% of SiAMT genes have introns. Motifs are relatively conserved short sequences shared by gene families, potentially serving as recognition sequences or functional proteins [52]. In this study, the *SiAMT7* and *SiAMT2* genes had the fewest motifs, likely explaining the functional diversity of AMT genes.

*Cis*-acting elements play an important regulatory role in gene expression [53]. This study predicted the functions of SiAMT genes by analyzing their *cis*-acting elements. The promoter region of SiAMT genes contained a variety of elements such as stress and hormone responses. Within the category of stress response elements, light-induced elements constituted the largest group. Among hormone response elements, the most abundant were those responsive to gibberellic acid. Furthermore, this study found that the promoter regions of the AMT gene families in both foxtail millet and soybean contain circadian control elements and anaerobic-responsive elements [40]. This result indicates that the transcriptional regulation of AMTs had already been established and remained highly conserved before the divergence of dicotyledonous and monocotyledonous plants, exhibiting evolutionary conservation. Their expression is strictly controlled by the circadian clock to achieve day-night coordination between nitrogen uptake and photosynthetic carbon production. The presence of anaerobic-responsive elements suggests that both crops have retained the molecular mechanism to respond to root hypoxia stress (e.g., waterlogging), enabling the dynamic regulation of ammonium uptake efficiency under soil flooding or other stress conditions to avoid metabolic disorders. This regulatory mode indicates that plants not only passively respond to environmental ammonium concentrations but also can anticipate environmental changes through endogenous circadian rhythms, actively optimizing the carbon/nitrogen balance.

Duplication events are a key driver of genome evolution, genetic system diversification, and biological diversity [54]. The expansion or contraction of gene family size is driven by environmental conditions as well as artificial selection. However, during the evolution of green foxtail to foxtail millet, the number of AMT genes did not change, which indicates that the evolution of AMT genes was relatively conservative. The uneven distribution of genes on each chromosome indicates the existence of genetic variation during the evolutionary process [55]. The positions of AMT genes on the chromosomes clearly showed the physical location distribution of each AMT gene in the genome. There is a tandem repeat on chromosome 5 of *SiAMT4* and *SiAMT5*, as well as a tandem repeat on chromosome 5 of *SvAMT4* and *SvAMT5*, which further indicates that the evolution of these genes was relatively conservative.

AMTs play key roles in ammonium transport, growth development, and environmental adaptation. The overexpression of rice *AMT1;1* enhances its resistance to rice sheath blight [56]. Studies have shown that the differentially expressed genes in Yugu 1 following treatment with 200 mM NaCl stress are involved in various metabolic, developmental, and stress response processes [57]. There is a certain correlation between salt stress and ammonium transporter-related genes [33]. This study found that, under 200 mM NaCl stress treatment, *SiAMT1*, *SiAMT2*, *SiAMT3*, *SiAMT5*, *SiAMT7*, and *SiAMT8* were all induced by salt stress. The expression patterns of *SiAMT1*, *SiAMT3*, and *SiAMT7* in the transcriptome data from different tissues were consistent with their expression patterns under salt stress. The growth environment of foxtail millet is influenced by multiple factors, such as nitrogen deficiency, salinity, and drought. Therefore, future research should pay more attention to the functions of these three genes in transporting ammonium ions.

In this study, the gene structure, *cis*-acting elements, and expression patterns of AMT gene family members in foxtail millet under salt stress were analyzed using bioinformatic methods. However, molecular biology experiments are still required in later stages to validate the specific molecular mechanisms of this gene family and its expression characteristics under interactions with other stress factors, in order to comprehensively understand the potential functions and mechanisms of this gene family.

## 5. Conclusions

In this study, a total of nine SiAMT genes were identified through genome-wide identification in foxtail millet. Their phylogenetic relationships, gene structures, conserved motifs, chromosomal locations, gene duplications, promoter *cis*-acting elements, and expression patterns induced by salt stress were comprehensively analyzed. The results show that the SiAMT family could be divided into three subfamilies, and the nine SiAMT genes were unevenly distributed across nine chromosomes. These conclusions establish a biological foundation for further studies on the gene functions of AMTs.

## Figures and Tables

**Table 1:** Analysis of gene information and physicochemical properties of AMT protein.

Gene Name	Gene ID	Protein Length	Molecular Weight (kDa)	Isoelectric Point (pI)	Instability Index	Hydropathicity	Subcellular Localization
*SiAMT1*	Seita.1G189700.1	479	51.91141	6.24	32.24	0.562	plas:11, vacu: 2, E.R.:1
*SiAMT2*	Seita.1G237300.1	491	51.78865	7.65	24.28	0.534	plas:8, vacu: 4, E.R.:2
*SiAMT3*	Seita.3G209900.1	489	51.73235	8.41	33.11	0.540	plas: 10, vacu: 3, E.R.:1
*SiAMT4*	Seita.5G368800.1	489	51.79242	8.82	34.81	0.517	plas: 11, vacu: 3
*SiAMT5*	Seita.5G368900.1	504	53.82384	7.08	34.21	0.499	plas: 10, vacu: 4
*SiAMT6*	Seita.5G395800.1	489	52.91538	6.25	31.35	0.420	plas: 12, vacu: 1, E.R.:1
*SiAMT7*	Seita.7G162400.1	495	52.30524	7.62	22.15	0.490	plas: 10, vacu: 2, E.R.:2
*SiAMT8*	Seita.9G019500.1	487	51.79834	7.59	33.47	0.539	plas: 12, vacu: 1, E.R.:1
*SiAMT9*	Seita.9G091900.1	465	49.46669	6.65	31.99	0.592	plas: 7, vacu: 4, E.R.:2, chlo: 1

## Data Availability

The original contributions presented in this study are included in the article; further inquiries can be directed to the corresponding authors.

## Supplementary Materials

The following supporting information can be downloaded at https://www.mdpi.com/article/10.3390/biology15090710/s1, Table S1: Primers for real-time qRT-PCR.

## References

[1] Chen K.E., Chen H.Y., Tseng C.S., Tsay Y.F. (2020). Improving nitrogen use efficiency by manipulating nitrate remobilization in plants. Nat. Plants.

[2] Crawford N.M., Forde B.G. (2002). Molecular and developmental biology of inorganic nitrogen nutrition. Arab. Book.

[3] Kissel D.E., Bock B.R., Ogles C.Z. (2020). Thoughts on acidification of soils by nitrogen and sulfur fertilizers. Agrosystems Geosci. Environ..

[4] Liu X., Zhang Y., Han W., Tang A., Shen J., Cui Z., Vitousek P., Erisman J.W., Goulding K., Christie P. (2013). Enhanced nitrogen deposition over China. Nature.

[5] Zhang W., Cao G., Li X., Zhang H., Wang C., Liu Q., Chen X., Cui Z., Shen J., Jiang R. (2016). Closing yield gaps in China by empowering smallholder farmers. Nature.

[6] Zeng H., Chen H., Zhang M., Ding M., Xu F., Yan F., Kinoshita T., Zhu Y. (2024). Plasma membrane H+-ATPases in mineral nutrition and crop improvement. Trends Plant Sci..

[7] Bloom A.J., Sukrapanna S.S., Warner R.L. (1992). Root respiration associated with ammonium and nitrate absorption and assimilation by barley. Plant Physiol..

[8] Gazzarrini S., Lejay L., Gojon A., Ninnemann O., Frommer W.B., von Wiren N. (1999). Three functional transporters for constitutive, diurnally regulated, and starvation-induced uptake of ammonium into Arabidopsis roots. Plant Cell.

[9] Li B., Zhao Y., Wang S., Zhang X., Wang Y., Shen Y., Yuan Z. (2021). Genome-wide identification, gene cloning, subcellular location and expression analysis of SPL gene family in *P. granatum* L.. BMC Plant Biol..

[10] Yuan L., Loqué D., Kojima S., Rauch S., Ishiyama K., Inoue E., Takahashi H., von Wirén N. (2007). The organization of high-affinity ammonium uptake in Arabidopsis roots depends on the spatial arrangement and biochemical properties of AMT1-type transporters. Plant Cell.

[11] Chiasson D.M., Loughlin P.C., Mazurkiewicz D., Mohammadidehcheshmeh M., Fedorova E.E., Okamoto M., McLean E., Glass A.D.M., Smith S.E., Bisseling T. (2014). Soybean *SAT1* (*Symbiotic Ammonium Transporter 1*) encodes a bHLH transcription factor involved in nodule growth and NH_4_^+^ transport. Proc. Natl. Acad. Sci. USA.

[12] Kronzucker H.J., Siddiqi M.Y., Glass A. (1996). Kinetics of NH4+ Influx in Spruce. Plant Physiol..

[13] Salvagiotti F., Cassman K., Specht J., Walters D., Weiss A., Dobermann A. (2008). Nitrogen uptake, fixation and response to fertilizer N in soybeans: A review. Field Crop. Res..

[14] Couturier J., Montanini B., Martin F., Brun A., Blaudez D., Chalot M. (2007). The expanded family of ammonium transporters in the perennial poplar plant. New Phytol..

[15] Javelle A., Morel M., Rodríguez-Pastrana B., Botton B., André B., Marini A., Brun A., Chalot M. (2003). Molecular characterization, function and regulation of ammonium transporters (Amt) and ammonium-metabolizing enzymes (GS, NADP-GDH) in the ectomycorrhizal fungus *Hebeloma cylindrosporum*. Mol. Microbiol..

[16] Hao D.L., Zhou J.Y., Yang S.Y., Qi W., Yang K.J., Su Y.H. (2020). Function and Regulation of Ammonium Transporters in Plants. Int. J. Mol. Sci..

[17] Konishi N., Ma J.F. (2021). Three polarly localized ammonium transporter 1 members are cooperatively responsible for ammonium uptake in rice under low ammonium condition. New Phytol..

[18] Kaiser B.N., Rawat S.R., Siddiqi M.Y., Masle J., Glass A.D. (2002). Functional analysis of an Arabidopsis T-DNA “knockout” of the high-affinity NH4+ transporter AtAMT1;1. Plant Physiol..

[19] Meier-Wagner J., Nolden L., Jakoby M., Siewe R., Krämer R., Burkovski A. (2001). Multiplicity of ammonium uptake systems in *Corynebacterium glutamicum*: Role of Amt and AmtB. Microbiology.

[20] Westengen O.T., Ring K.H., Berg P.R., Brysting A.K. (2014). Modern maize varieties going local in the semi-arid zone in Tanzania. BMC Evol. Biol..

[21] Liu Y., von Wirén N. (2017). Ammonium as a signal for physiological and morphological responses in plants. J. Exp. Bot..

[22] Maniero R.A., Koltun A., Vitti M., Factor B.G., de Setta N., Câmara A.S., Lima J.E., Figueira A. (2023). Identification and functional characterization of the sugarcane (*Saccharum* spp.) AMT2-type ammonium transporter ScAMT3;3 revealed a presumed role in shoot ammonium remobilization. Front. Plant Sci..

[23] Zhang W., Lin L., Wang T., Chen M., Song B., Sun W. (2022). Genome-wide identification of AMT2-type ammonium transporters reveal that CsAMT2.2 and CsAMT2.3 potentially regulate NH4+ absorption among three different cultivars of *Camellia sinensis*. Int. J. Mol. Sci..

[24] Von Wirén N., Lauter F.-R., Ninnemann O., Gillissen B., Walch-Liu P., Engels C., Jost W., Frommer W.B. (2000). Differential regulation of three functional ammonium transporter genes by nitrogen in root hairs and by light in leaves of tomato. Plant J..

[25] Meng Y.-Y., Wang N., Zhang H.-Y., Xu R., Si C.-C. (2023). Genome-Wide Analysis of Sweet Potato Ammonium Transporter (AMT): Influence on Nitrogen Utilization, Storage Root Development and Yield. Int. J. Mol. Sci..

[26] Wu X.X., Yuan D.P., Chen H., Kumar V., Kang S.M., Jia B., Xuan Y.H. (2022). Ammonium transporter 1 increases rice resistance to sheath blight by promoting nitrogen assimilation and ethylene signalling. Plant Biotechnol. J..

[27] Jiang J., Zhao J., Duan W., Tian S., Wang X., Zhuang H., Fu J., Kang Z. (2019). TaAMT2;3a, a wheat AMT2-type ammonium transporter, facilitates the infection of stripe rust fungus on wheat. BMC Plant Biol..

[28] Chen X., Wang R., Mao X., Dong M., Chen L., Li Y., Sun H. (2026). VcAMT14 Enhances Ammonium Uptake in Blueberries During Mycorrhizal Symbiosis. Plant Cell Environ..

[29] Simon-Rosin U., Wood C., Udvardi M.K. (2003). Molecular and cellular characterisation of LjAMT2;1, an ammonium transporter from the model legume *Lotus japonicus*. Plant Mol. Biol..

[30] Guether M., Neuhäuser B., Balestrini R., Dynowski M., Ludewig U., Bonfante P. (2009). A mycorrhizal-specific ammonium transporter from *Lotus japonicus* acquires nitrogen released by arbuscular mycorrhizal fungi. Plant Physiol..

[31] Breuillin-Sessoms F., Floss D.S., Gomez S.K., Pumplin N., Ding Y., Levesque-Tremblay V., Noar R.D., Daniels D.A., Bravo A., Eaglesham J.B. (2015). Suppression of Arbuscule Degeneration in Medicago truncatula phosphate transporter4 Mutants Is Dependent on the Am-monium Transporter 2 Family Protein AMT2;3. Plant Cell.

[32] Cai Z., Yu T., Tan W., Zhou Q., Liu L., Nian H., Lian T. (2024). GmAMT2.1/2.2-dependent ammonium nitrogen and metabolites shape rhizosphere microbiome assembly to mitigate cadmium toxicity. npj Biofilm. Microbiomes.

[33] Ma L., Qin D.-B., Sun L., Zhang K., Yu X., Dang A.-K., Hou S., Zhao X., Yang Y., Wang Y. (2025). SALT OVERLY SENSITIVE2 and AMMONIUM TRANSPORTER1;1 contribute to plant salt tolerance by maintaining ammonium uptake. Plant Cell.

[34] Ma Z., Yang X., Zhang C., Sun Y., Jia X. (2016). Early millet use in West Liaohe area during early-middle Holocene. Sci. China Earth Sci..

[35] Peng R., Zhang B. (2021). Foxtail millet: A new model for C4 plants. Trends Plant Sci..

[36] Sachdev N., Goomer S., Singh L.R. (2021). Foxtail millet: A potential crop to meet future demand scenario for alternative sustainable protein. J. Sci. Food Agric..

[37] Bennetzen J.L., Schmutz J., Wang H., Percifield R., Hawkins J., Pontaroli A.C., Estep M., Feng L., Vaughn J.N., Grimwood J. (2012). Reference genome sequence of the model plant Setaria. Nat. Biotechnol..

[38] Diao X., Schnable J., Bennetzen J.L., Li J. (2014). Initiation of Setaria as a model plant. Front. Agric. Sci. Eng..

[39] Yang Z., Zhang H., Li X., Shen H., Gao J., Hou S., Zhang B., Mayes S., Bennett M., Ma J. (2020). A mini foxtail millet with an Arabidopsis-like life cycle as a C4 model system. Nat. Plants.

[40] Yang W., Dong X., Yuan Z., Zhang Y., Li X., Wang Y. (2023). Genome-wide identification and expression analysis of the ammonium transporter family genes in Soybean. Int. J. Mol. Sci..

[41] Wu Z., Gao X., Zhang N., Feng X., Huang Y., Zeng Q., Wu J., Zhang J., Qi Y. (2021). Genome-wide identification and transcriptional analysis of ammonium transporters in Saccharum. Genomics.

[42] Dai J., Han P., Walk T.C., Yang L., Chen L., Li Y., Gu C., Liao X., Qin L. (2023). Genome-wide identification and characterization of ammonium transporter (AMT) genes in Rapeseed (*Brassica napus* L.). Genes.

[43] Fang L., Wang M., Chen X., Zhao J., Wang J., Liu J. (2023). Analysis of the AMT gene family in chili pepper and the effects of arbuscular mycorrhizal colonization on the expression patterns of CaAMT2 genes. BMC Genom..

[44] Wu X., Yang H., Qu C., Xu Z., Li W., Hao B., Yang C., Sun G., Liu G. (2015). Sequence and expression analysis of the AMT gene family in poplar. Front. Plant Sci..

[45] Gu R., Duan F., An X., Zhang F., von Wirén N., Yuan L. (2013). Characterization of AMT-mediated high-affinity ammonium uptake in roots of maize (*Zea mays* L.). Plant Cell Physiol..

[46] Li T., Liao K., Xu X., Gao Y., Wang Z., Zhu X., Jia B., Xuan Y. (2017). Wheat ammonium transporter (AMT) gene family: Diversity and possible role in host-pathogen interaction with stem rust. Front. Plant Sci..

[47] Li B.-Z., Merrick M., Li S.-M., Li H.-Y., Zhu S.-W., Shi W.-M., Su Y.-H. (2009). Molecular basis and regulation of ammonium transporter in rice. Rice Sci..

[48] Xia Y., Liu Y., Zhang T., Wang Y., Jiang X., Zhou Y. (2022). Genome-wide identification and expression analysis of ammonium transporter 1 (AMT1) gene family in cassava (Manihot esculenta Crantz) and functional analysis of MeAMT1;1 in transgenic Arabidopsis. 3 Biotech.

[49] He Q., Tang S., Zhi H., Chen J., Zhang J., Liang H., Alam O., Li H., Zhang H., Xing L. (2023). A graph-based genome and pan-genome variation of the model plant Setaria. Nat. Genet..

[50] Hernandez-Garcia C.M., Finer J.J. (2014). Identification and validation of promoters and cis-acting regulatory elements. Plant Sci..

[51] Jo B.-S., Choi S.S. (2015). Introns: The functional benefits of introns in genomes. Genom. Inform..

[52] Morello L., Breviario D. (2008). Plant spliceosomal introns: Not only cut and paste. Curr. Genom..

[53] Kong F., Wang J., Cheng L., Liu S., Wu J., Peng Z., Lu G. (2012). Genome-wide analysis of the mitogen-activated protein kinase gene family in *Solanum lycopersicum*. Gene.

[54] Gu Z., Steinmetz L.M., Gu X., Scharfe C., Davis R.W., Li W.-H. (2003). Role of duplicate genes in genetic robustness against null mutations. Nature.

[55] Paterson A.H., Wendel J.F., Gundlach H., Guo H., Jenkins J., Jin D., Llewellyn D., Showmaker K.C., Shu S., Udall J. (2012). Repeated polyploidization of Gossypium genomes and the evolution of spinnable cotton fibres. Nature.

[56] Li D., Kumar V., Xuan Y. (2024). Ammonium transporter 1 promotes resistance to sheath blight in rice without yield loss. Agrobiodiversity.

[57] Man X., Tang S., Meng Y., Gong Y., Chen Y., Wu M., Jia G., Liu J., Diao X., Cheng X. (2025). Convergent and divergent signaling pathways in C3 rice and C4 foxtail millet crops in response to salt stress. J. Integr. Agric..

