# Supplementary material for: The Genome-Wide Identification and Expression Analysis of the AMT Gene Family in Foxtail Millet
Source: Biology (Basel). 2026 Apr 30;15(9):710. doi: 10.3390/biology15090710 (PMC13162773; doi:10.3390/biology15090710)
Supplement: Supplementary file 1 [file biology-15-00710-s001.zip › biology-4244715-supplementary.pdf]

Table S1 Primers for real-time qRT-PCR

Gene name	Gene ID	Forward Prime (5'-3')	Reverse Prime (3'-5')
SiAMT1	Seita.1G189700.1	CTGCAACCTCTTCCTCTCCA	CCAGGCAATGACGAAGAGTG
SiAMT2	Seita.1G237300.1	CTCGACGTACCTGCTCTTCT	CACGTTGGTGAGCATGATGT
SiMAT3	Seita.3G209900.1	GGCTCCTCTTCTCCTACACC	ATCAGCAGGATGTTGTTCGG
SiAMT4	Seita.5G368800.1	GATACGTCGTCCACCTCTCC	GGCACTGACATTGGTGTTGA
SiAMT5	Seita.5G368900.1	TGCCTCGACGTCATCTTCTT	GACATGCCCATCAGTATCGC
SiAMT6	Seita.5G395800.1	TTCAAGAAGCCCTCCGTCAT	CACCACCATCATCGTGAACC
SiAMT7	Seita.7G162400.1	ACATCATGCTCACCAACGTG	CGAAGAACTGCTTCCCGATG
SiAMT8	Seita.9G019500.1	GACGGTGGTGTACTTCCAGT	AAGATCATCCACGCCAGGAA
SiAMT9	Seita.9G091900.1	GTCATCCACCTCTCCTCTGG	GGATGTTGTTAGGCGGGAAC
SiActin	AF288226	GGCAAACAGGGAGAAGATGA	GAGGTTGTCGGTAAGGTCACG
